# Complete histologic response to neoadjuvant pembrolizumab in high-risk stage IIC melanoma of the nasal ala: A case report

**DOI:** 10.1016/j.jdcr.2026.08.028

**Published:** 2026-08-25

**Authors:** Emma Clochard, Julien Anriot, Mona Amini-Adle, Eve-Marie Neidhardt

**Affiliations:** a Department of Dermatology, Centre Léon Bérard, Lyon, France; b Department of Oncology, Centre Léon Bérard, Lyon, France

**Keywords:** immune checkpoint inhibitor, melanoma, neoadjuvant, pembrolizumab

## Introduction

Cutaneous melanoma management relies primarily on surgical excision with adequate margins.^1^ In locally advanced cases, achieving these margins can cause significant functional and aesthetic morbidity, particularly on the face. We report a case of locally advanced melanoma of the left nasal ala demonstrating remarkable response to neoadjuvant pembrolizumab, thereby avoiding potentially disfiguring surgery.

## Case report

A 52-year-old man with a history of hypertension presented in February 2025 with an exophytic lesion on his left nasal ala (Fig 1, *A*). Biopsy revealed invasive melanoma with ulceration, Clark level IV minimum, Breslow thickness 4.4 mm, and BRAF wild-type status.

**Fig 1:**
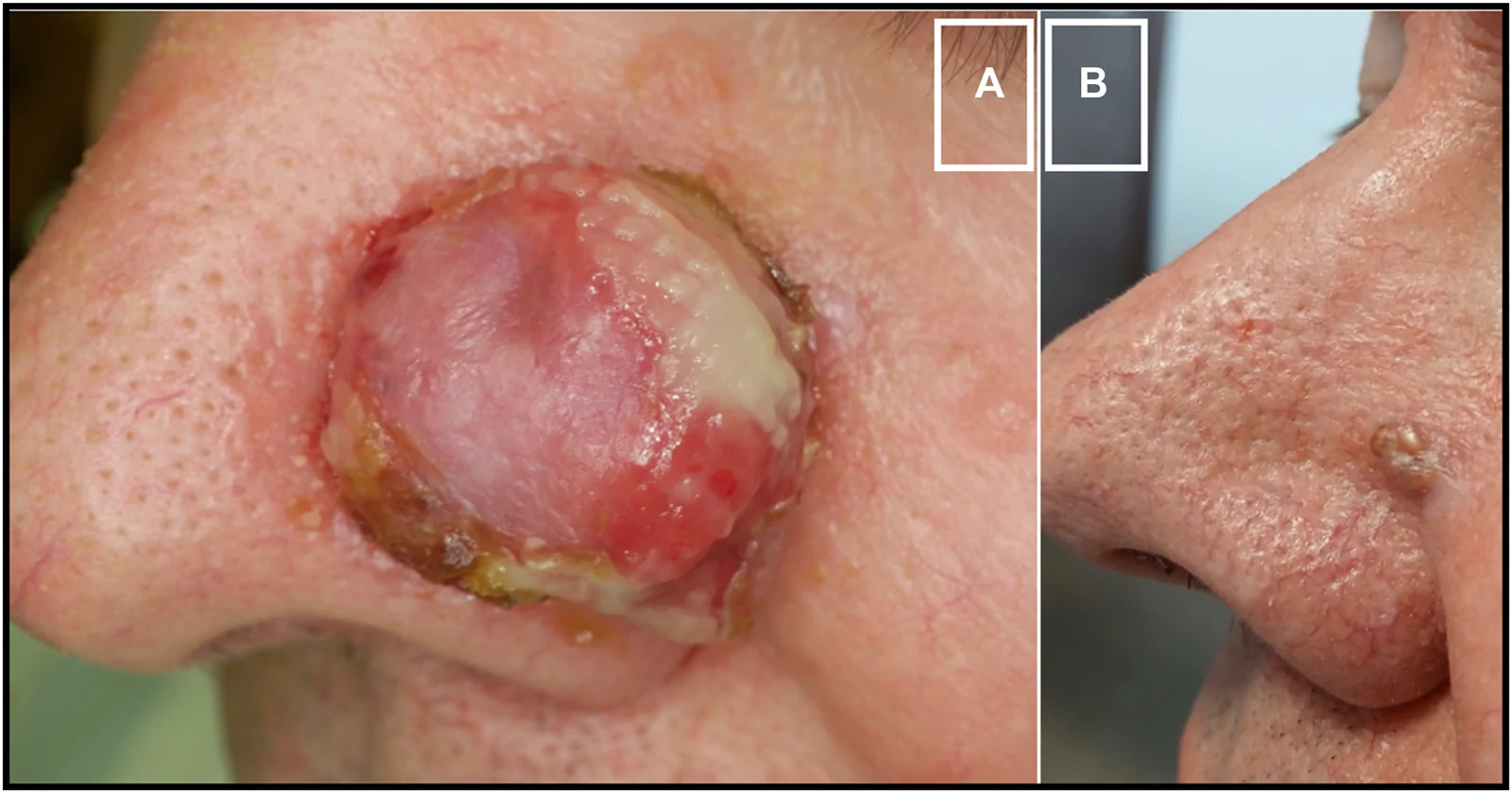
Melanoma of the left nasal ala **(A)** before treatment and **(B)** after 3 pembrolizumab cycles.

Positron emission tomography–computed tomography and brain imaging demonstrated no distant metastases, with hypermetabolism confined to the primary site.

Standard management for this stage IIC melanoma would include wide local excision with 2 cm margins and potential sentinel lymph node biopsy, followed by adjuvant anti-PD-1 therapy for 1 year,^1^ a particularly mutilating intervention at this facial location.

Following multidisciplinary tumor board discussion and comprehensive patient counseling, we initiated off-label neoadjuvant pembrolizumab (200 mg intravenously every 3 weeks for 3 cycles) to reduce tumor burden and enable less disfiguring surgery. This decision was motivated by the high risk of functional and aesthetic impairment, alongside emerging evidence of neoadjuvant pembrolizumab efficacy, notably from the NADINA and SWOG S1801 trials in stage III disease.^2^^,^^3^

After 3 cycles, a major clinical response occurred with near-complete lesion regression (Fig 1, *B*). Biopsy of the residual area confirmed a complete pathologic response without significant viable tumor cells (Supplementary Fig 1, available via Mendeley at https://data.mendeley.com/datasets/nvt5wsgswy/1). No further surgery was proposed.

Treatment was well tolerated with only grade 1 fatigue. After the seventh infusion, the patient developed immune-mediated thyroiditis, which was controlled with levothyroxine replacement therapy. The patient received a total of 9 pembrolizumab infusions.

Treatment was discontinued at the end of August 2025 because of a gradual elevation in serum creatinine level (grade 1) associated with elevated creatine phosphokinase level (grade 2) and intolerable fatigue (grade 2).

Positron emission tomography–computed tomography performed in October 2025 revealed right axillary lymphadenopathy, which was biopsied and confirmed as benign reactive lymphoid tissue. To date, the patient remains in complete metabolic remission 11 months after initiation of immunotherapy (Fig 2).

**Fig 2:**
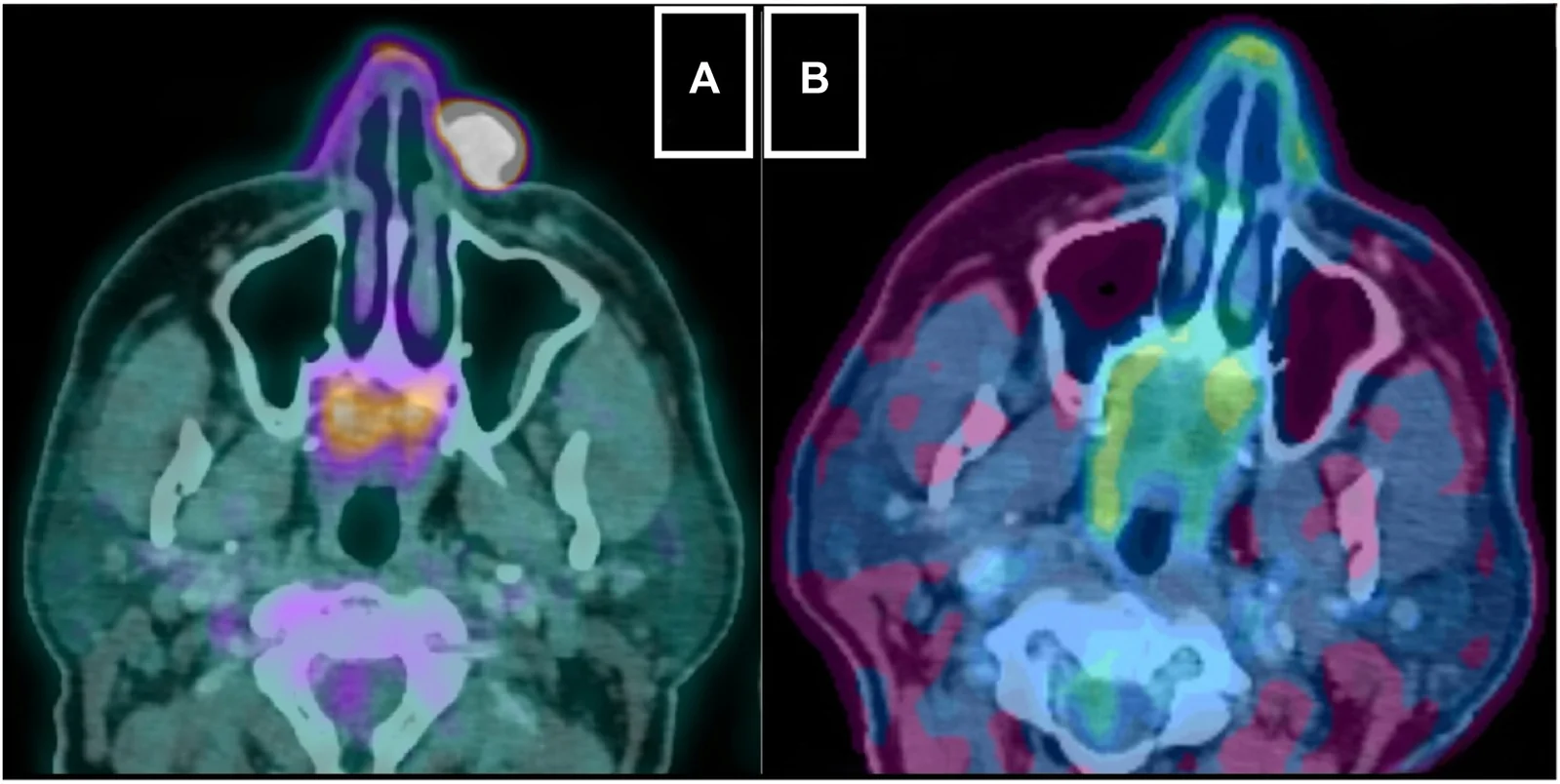
FDG Positron emission tomography–computed tomography imaging of the left nasal ala region demonstrating metabolic evolution under immunotherapy, **(A)** January 2025, before treatment initiation, and **(B)** January 2026, after 9 infusions.

## Discussion

Although the benefit of neoadjuvant immunotherapy is now established in stage III melanoma through the NADINA and SWOG S1801 trials, these data derive primarily from patients with clinically detectable nodal disease.

Our case suggests that this strategy may also benefit carefully selected patients with high-risk primary melanomas in which upfront surgery would result in substantial functional or aesthetic compromise, illustrating how treatment paradigms established in more advanced disease may be judiciously extrapolated to select cases of locally advanced primary tumors, bal ancing oncologic outcomes with quality-of-life considerations. This case aligns with other reports.^4^

The results of the NCT03757689 trial^5^ will help define optimal patient selection criteria, treatment duration, and long-term outcomes for this approach in high-risk primary melanomas.

### Declaration of generative AI and AI-assisted technologies in the writing process

During the preparation of this work the author(s) used Claude in order to correct the grammar. After using this tool/service, the author(s) reviewed and edited the content as needed and take(s) full responsibility for the content of the publication.

## Conflicts of interest

None disclosed.

## References

[1] Garbe C., Amaral T., Peris K. (2025). European consensus-based interdisciplinary guideline for melanoma. Part 2: treatment—Update 2024. Eur J Cancer.

[2] Blank C.U., Lucas M.W., Scolyer R.A. (2024). Neoadjuvant nivolumab and ipilimumab in resectable stage III melanoma. N Engl J Med.

[3] Patel S.P., Othus M., Chen Y. (2023). Neoadjuvant-adjuvant or adjuvant-only pembrolizumab in advanced melanoma. N Engl J Med.

[4] Amiot M., Battistella M., Bezier M., Lebbé C., Baroudjian B. (2024). Efficacy of neoadjuvant pembrolizumab in a primary cutaneous stage II non-operated melanoma. EJC Skin Cancer.

[5] Neoadjuvant PD-1 blockade in patients with stage IIB/C melanoma. ClinicalTrials.gov identifier: NCT03757689. NCT03757689.

